# Digital Health Literacy and Its Association With Sociodemographic Characteristics, Health Resource Use, and Health Outcomes: Rapid Review

**DOI:** 10.2196/46888

**Published:** 2024-07-26

**Authors:** Eva Yuen, Natalie Winter, Feby Savira, Catherine E Huggins, Lemai Nguyen, Paul Cooper, Anna Peeters, Kate Anderson, Rahul Bhoyroo, Sarah Crowe, Anna Ugalde

**Affiliations:** 1 School of Nursing and Midwifery Deakin University Burwood Australia; 2 Centre for Quality and Patient Safety Research Institute for Health Transformation Deakin University Burwood Australia; 3 Monash Health Clayton Australia; 4 Global Centre for Preventative Health and Nutrition Institute for Health Transformation Deakin University Burwood Australia; 5 Deakin Health Economics Institute for Health Transformation Deakin University Burwood Australia; 6 School of Health and Social Development Deakin University Burwood Australia; 7 Department of Information Systems and Business Analytics Deakin Business School Deakin University Burwood Australia; 8 School of Medicine Deakin University Burwood Australia; 9 Institute for Health Transformation Deakin University Burwood Australia; 10 School of Computing Technologies STEM College RMIT University Melbourne Australia; 11 Western Victoria Primary Health Network Geelong Australia

**Keywords:** digital health literacy, eHealth literacy, health literacy, digital health, web-based database, health information

## Abstract

**Background:**

Digital health literacy has emerged as a critical skill set to navigate the digital age.

**Objective:**

This review sought to broadly summarize the literature on associations between digital health literacy and (1) sociodemographic characteristics, (2) health resource use, and (3) health outcomes in the general population, patient groups, or parent or caregiver groups.

**Methods:**

A rapid review of literature published between January 2016 and May 2022 was conducted through a search of 4 web-based databases. Articles were included on the basis of the following keywords: “measured digital health literacy,” “digital literacy,” “ehealth literacy,” “e-health literacy,” “electronic health literacy,” or “internet health literacy” in adult populations; participants were from countries where English was the primary language; studies had to be cross-sectional, longitudinal, prospective, or retrospective, and published in English.

**Results:**

Thirty-six articles met the inclusion criteria. Evidence on the associations between digital health literacy and sociodemographic characteristics varied (27/36, 75% included studies), with higher education (16/21, 76.2% studies that examined the association) and younger age (12/21, 57.1% studies) tending to predict higher digital health literacy; however, other studies found no associations. No differences between genders were found across the majority of studies. Evidence across ethnic groups was too limited to draw conclusions; some studies showed that those from racial and ethnic minority groups had higher digital health literacy than White individuals, while other studies showed no associations. Higher digital health literacy was associated with digital health resource use in the majority of studies (20/36, 55.6%) that examined this relationship. In addition, higher digital health literacy was also associated with health outcomes across 3 areas (psychosocial outcomes; chronic disease and health management behaviors; and physical outcomes) across 17 included studies (17/36, 47.2%) that explored these relationships. However, not all studies on the relationship among digital health literacy and health resource use and health outcomes were in the expected direction.

**Conclusions:**

The review presents mixed results regarding the relationship between digital health literacy and sociodemographic characteristics, although studies broadly found that increased digital health literacy was positively associated with improved health outcomes and behaviors. Further investigations of digital health literacy on chronic disease outcomes are needed, particularly across diverse groups. Empowering individuals with the skills to critically access and appraise reliable health information on digital platforms and devices is critical, given emerging evidence that suggests that those with low digital health literacy seek health information from unreliable sources. Identifying cost-effective strategies to rapidly assess and enhance digital health literacy capacities across community settings thus warrants continued investigation.

## Introduction

As health technologies evolve, digital devices, health-related apps, and web-based portals are increasingly used to deliver and access medical information and health care services [1]. While such technologies can be a gateway to health information and support [2], research also predicts a “digital divide” in which an individual’s sociodemographic characteristics (eg, age, education, and income) influence their effective engagement with digital health information [3]. Digital health literacy has emerged as a vital skill set to navigate health care in the digital age [4]. Digital health literacy has been described as an extension of eHealth literacy, which captures the skills to seek, find, understand, and critically appraise health information from electronic sources to manage one’s own health [5,6]. Digital health literacy has been posited to expand on the definition by emphasizing the individual as both an active participant and a distributer of digital health information, not just a passive receiver [6]. Digital health literacy has skills unique to health literacy, including computer literacy, media literacy, and critical appraisal skills to identify and evaluate reliable information and resources [1]. The importance of digital health literacy is increasingly recognized for its role in optimal individual and population health [4] and critical to limiting health inequalities [7].

Reviews have reported associations between digital health literacy and health outcomes across specific populations. Among people with long-term conditions, higher digital health literacy was predominantly associated with greater health-promoting behaviors [8]. A recent review identified that both older adults with cancer and their carers reported low digital health literacy and decreased confidence in appraising digital health information, with barriers identified including low socioeconomic status, poor digital access, and lack of familiarity and use [9]. Several reviews have examined the impact of digital health literacy on health outcomes in specific populations of interest (eg, older adults [9], those with long-term conditions [8], college students [10], and underserved [11] or vulnerable populations [12]). However, existing evidence on the relationships between digital health literacy, health outcomes, and sociodemographic characteristics across broad population groups has not been synthesized to date. Current literature on digital health literacy has focused on definitions and scales; its associations with health outcomes; the digital divide; and influencing factors of health literacy [13]. A review of associations between (1) sociodemographic characteristics, (2) health resource use, and (3) health outcomes in the general population, patient groups, or parent or caregiver groups is currently lacking. This information is critical to inform the development and implementation of digital health strategies to improve digital health literacy in communities with the highest need. This review sought to broadly summarize the literature on associations between digital health literacy and (1) sociodemographic characteristics, (2) health resource use, and (3) health outcomes.

## Methods

A rapid review was undertaken following the principles of a systematic review [14]; however, with some simplification of steps to ensure a timely and accurate synthesis of evidence to inform the development of a digital health strategy for implementation across community settings. Given the rapid nature of the review, the review and protocol were not registered with an international register.

A search for peer-reviewed publications was undertaken within the CINAHL, PsycINFO, MEDLINE, and Embase databases in May 2022. English language articles published between January 2016 and May 2022 were included. Given then rapid changes in technology and digital engagement observed in the health field, we sought to comprehensively review contemporary evidence from the preceding 5 years only. Additional searches were conducted in June 2022 using Google Scholar and by handsearching the reference lists of included papers to ensure that all relevant literature were captured in the review. Search terms synonyms were “digital health literacy,” “e-health literacy,” “electronic health literacy,” “internet literacy,” “internet health literacy,” and “digital literacy.” Key search terms are detailed in Multimedia Appendix 1. For the purposes of the review, studies that examined electronic health literacy or eHealth literacy were included given similarities in definitions of these concepts [13]. The inclusion and exclusion criteria are summarized in Table 1. In brief, studies that examined digital health literacy using a validated measure of digital health literacy in the general population, patient groups, or parent or caregiver groups were eligible for inclusion. Articles were excluded if they focused exclusively on college students, since a review of this group has already been conducted [10].

**Table 1:** Inclusion and exclusion criteria.

Inclusion	Exclusion
Assesses digital health literacy in the general population, patients, parents, or caregivers	Study population younger than 18 years
Sample included individuals from Australia, New Zealand, the United States, Canada, the United Kingdom, or Ireland	Primary language of country is not English
Quantitative study	Qualitative study, literature or systematic review, commentary, conference abstract, opinion piece, protocol, or thesis
Published in 2016 onwards	Non–health care–related (eg, high school education)
—^a^	Not focused on patients or general population (eg, digital health literacy of health providers)
—	Focusses on digital health literacy of interventions, programs, etc
—	Assesses psychometric properties of a digital health literacy measure
—	Measures digital health literacy using a single item or proxy measure

^a^Not applicable.

The search results were imported into a review management dashboard, Covidence, to allow for simultaneous screening between reviewers. Four reviewers (EY, NW, FS, and FT) independently screened titles and abstracts, and the full texts were screened by 5 reviewers (EY, NW, FS, FT, and SR). At both screening stages, each paper was assessed by 2 reviewers, with discrepancies resolved through discussion. The screening process was reported following the PRISMA (Preferred Reporting Items for Systematic Reviews and Meta-Analyses) guidelines [15].

For studies included in the review, data were extracted on (1) study characteristics (eg, author, publication year, country, study design, sample size, participant characteristics, measures used to assess digital health literacy, and health literacy scores); (2) health outcomes associated with digital health literacy; and (3) sociodemographic characteristics associated with digital health literacy. Given the rapid nature of the review to inform strategy, data extraction for each article was conducted by 1 author only (EY, NW, or FS), with data extracted presented to the research team for review. Results were narratively synthesized to address the 3 aims and to group findings into identified themes.

## Results

### Description of Included Studies

**Figure 1:**
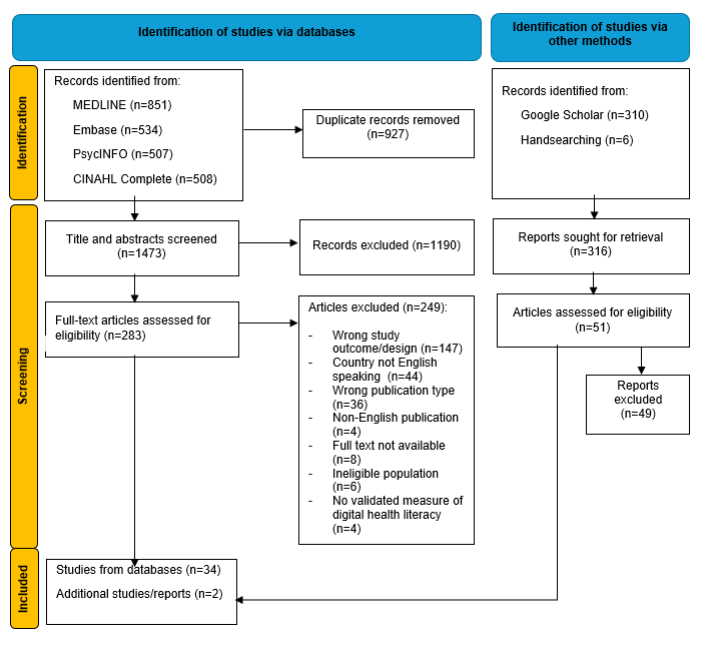
PRISMA Flowchart.

Of the 1473 articles, 36 met the inclusion criteria (Figure 1). Studies are summarized in Multimedia Appendix 2. Twenty-five studies were from the United States, 4 studies from Australia [16-19], 4 from Canada [20-23], 1 from the United Kingdom [24], and 1 cross-cultural study that included people from Australia and India [25]. Studies included 34 cross-sectional survey designs, 1 mixed methods study [26], and 1 randomized controlled trial [27]. Sample sizes ranged from 22 [28] to 3258 [29] participants. The majority of studies (32/36, 88.9%) assessed digital health literacy using the eHealth Literacy Scale [30]. Across studies, variations on how digital health literacy scores were reported; some used a mean score (with higher scores indicating higher digital health literacy) and other studies used cutoff scores to determine those in high compared with low digital health literacy categories.

Five distinct population groups were identified across the studies. These included the general population, including older adults, patient groups, minority populations, and caregivers. Patient groups included people with HIV, cancer, chronic obstructive pulmonary disease (COPD), spinal injury, kidney disease, bipolar disorder, otolaryngological disease, orthopedic trauma, cardiovascular risk, diabetes, and transplant recipients. Minority groups included African Americans, Southeast Asians, adults in rural or remote regions, low-income groups, transgender and gender diverse, and young men who have sex with men. Caregivers included those of children with special health care needs, men with prostate cancer, and pediatric inpatients with subacute health conditions.

### Associations Between Digital Health Literacy and Sociodemographic Characteristics

**Figure 2:**
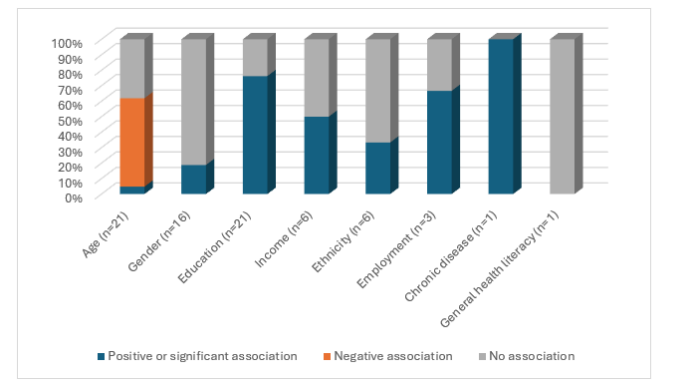
Digital health literacy and associations with sociodemographic characteristics. Positive association: higher digital health literacy associated with higher outcome of characteristics (eg, higher digital health literacy associated with higher income); negative association: higher digital health literacy associated with lower characteristics (eg, higher digital health literacy associated with younger age).

Twenty-seven (75%) studies were identified as examining associations between digital health literacy and sociodemographic characteristics, with varied findings. These are shown in Table 2 and Figure 2 and discussed in the following sections.

**Table 2:** Digital health literacy and associations with sociodemographic characteristics (N=27).

Sociodemographic characteristics and association with digital health literacy			Articles in which the finding occurred, n										
			Patient group		General population		Minority group		Older adults		Caregivers		Total
**Age (years)**													
	Negative	5		3		1		1		2		12	
	Positive	1		—^a^		—		—		—		1	
	None	5		—		1		2		—		8	
**Gender^b^**													
	Significant	1		1		1		—		—		3	
	None	7		3		—		3		—		13	
**Education**													
	Positive	7		3		1		3		2		16	
	None	3		1		—		1		—		5	
**Income**													
	Positive	1		1		—		—		1		3	
	None	2		—		—		1		—		3	
**Race/ethnicity**													
	Positive	—		2		—		—		—		2	
	None	3		—		—		—		1		4	
**Employment**													
	Positive	1		1		—		—		—		2	
	None	—		—		—		1		—		1	
**No chronic disease**													
	Positive	—		—		1		—		—		1	
**General health literacy**													
	None	—		—		—		1		—		1	
**Marital status**													
	None	3		1		—		2		—		6	
**Languages spoken**													
	None	1		1		—		—		—		2	
**Socioeconomic status**													
	None	1		—		—		1		—		2	
**Rural/urban**													
	None	1		—		—		—		—		1	

^a^Not applicable.

^b^Women had higher digital health literacy.

#### Age

Twenty-one studies (21/36, 58.3%) explored associations between age and levels of digital health literacy with mixed results. Twelve studies (12/21, 57.1%) reported a negative association between age and digital health literacy, with older people more likely to have lower digital health literacy than their younger counterparts [16,19-21,24-26,31-35]. Negative associations between age and digital health literacy were found in the general population (3/21, 14.3%) [16,25,32], as well as the following specific population studies: Southeast Asian adults in Canada [21]; older adults [19,20]; people with chronic kidney disease [26], breast cancer [24], or cardiovascular risk [19]; and caregivers [34,35]. One study that used the eHealth Literacy Questionnaire [16] found that age was negatively associated with 4 of 7 digital health literacy subscales (ability to engage with digital services; using technology to process health information; motivated to engage with digital health services; and digital services that suit individual needs).

In contrast, studies (1/21, 4.8%) found a positive significant association between age and digital health literacy in people with bipolar disorder, where older age significantly predicted higher digital health literacy scores [36]. Notably, the mean age in this sample was less than that in other studies, and more than three-quarters had completed some form of higher education. Furthermore, studies (8/21, 38.1%) reported no associations between age and digital health literacy across patient [17,23,37-39], rural [22], and older [40,41] populations.

#### Gender

Digital health literacy and gender were reported in studies (16/36, 44.4%), with most (13/16, 81.3%) finding no gender differences [16,19,20,23,26,31-33,36,39-42]. The 3 studies that found that women were more likely to perceive themselves as having higher digital health literacy than men were from the general population in the United States [43], adults from rural communities in British Columbia with a population of 12,000 [22], and among otolaryngology patients [37].

#### Education

Twenty-one (21/36, 58.3%) studies examined associations between digital health literacy and education levels, with two-thirds (16/21, 76.2%) finding higher digital health literacy positively associated with higher education levels [16,18-21,24,31-36,39,41,44,45]. Five studies (5/16, 31.3%) reported no associations between digital health literacy and education, including 4 studies of patient groups [21,28,29], 1 general population study [25], and 1 study of older adults [32]. Across these studies, large proportions of participants had greater than high school education levels (35%-84.8%).

#### Income

Six studies (6/36, 28.6%) examined associations between digital health literacy and income, with half reporting no associations in people living with chronic illness [19,23] or those in a general population [41]. By contrast, the 3 studies that found significant positive associations between higher digital health literacy and income reported these findings in people with chronic illness [26], among caregivers [35], and in minority populations [32].

#### Ethnicity

Six studies (6/36, 16.7%) examined associations between digital health literacy and ethnicity, with 2 reporting differences between ethnicity groups [25,32]. Black or African Americans had higher digital health literacy than their Caucasian counterparts in a stratified US sample [32]. Australians had higher digital health literacy than Indians in a cross-cultural study [25]. Conversely, 4 studies found no differences in digital health literacy levels across ethnicity groups among those with chronic illness [26], spinal cord injury [38], cancer survivors [24], or caregivers of people with prostate cancer [35].

#### Employment

Of the 3 studies (3/36, 8.3%) that examined associations between digital health literacy and employment, 1 patient group study (spinal cord injury [23]) and 1 general population study [43] found higher digital health literacy among those who were employed versus those who were unemployed. By contrast, another study found no differences in employment groups and digital health literacy in an older US sample [40].

#### Chronic Disease or Comorbidities

One study (1/36, 2.7%) reported small but significant findings for higher digital health literacy in people with no chronic conditions, compared with those with a chronic condition, in a sample of South Asians in Canada [21].

#### General Health Literacy

Only 1 study (1/36, 2.7%) examined associations between digital health literacy and general health literacy, finding no associations in an older adult sample [40].

#### Marital Status, Language Spoken, Socioeconomic Status, Household Size, Rural or Urban Location, or Country of Birth

No associations between digital health literacy and the following sociodemographic characteristics were found: marital status [17,25,38,41,46,47], language spoken [16,20], socioeconomic status or deprivation [24,40], household size [40], or rural or urban location [24].

### Associations Between Digital Health Literacy and Health Resource Navigation

**Figure 3:**
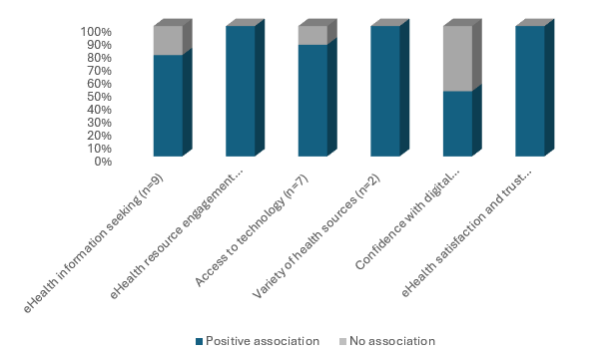
Digital health literacy and associations with health resource navigation.

Twenty studies (20/36, 55.6%) examined associations between digital health literacy and health resource navigation distributed across all participant categories. Categories of health information engagement included eHealth information seeking; eHealth behaviors; access to and use of e-resources; use of health information sources; and eHealth satisfaction, and are shown in Table 3, Figure 3, and in the following sections.

**Table 3:** Digital health literacy and digital health resource navigation (N=17).

Health outcomes	Association with digital health literacy	Articles in which association occurred, n					
		Patient group	General population	Minority group	Older adults	Caregivers	Total
eHealth information seeking	Positive	1	2	2	2	—^a^	7
eHealth information seeking	None	—	—	2	—	—	2
eHeath resource engagement	Positive	2	1	2	1	1	7
Access to and use of technology	Positive	1	1	2	1	2	7
Use of a variety of health information sources	Positive	—	—	1	1	—	2
Confidence or comfort with using digital resources	Positive	—	—	—	1	—	1
Confidence or comfort with using digital resources	None	—	1	—	—	—	1
eHealth satisfaction and trust	Positive	—	1	1	—	1	3

^a^Not applicable.

#### Digital Health Information Seeking

Nine studies (9/36, 25%) examined digital health literacy and digital health information seeking, and of these, 7 showed that higher digital health literacy was significantly positively associated with e-information seeking. These included studies of general populations [47,48], middle-aged to older adults [41,49], minority populations [18,21], and of people with HIV [50]. In addition, higher digital health literacy was associated with greater exposure to medical and health websites in a US general population [48] and among Southeast Asians in Canada [21]. Digital health literacy was also associated with eHealth information consumerism in older adults [41].

By contrast, 2 studies (2/36, 5.6%) found no associations between digital health literacy and eHealth information–seeking behaviors. In a sample of older Hispanic people in the United States, digital health literacy was not associated with use of, nor willingness to use the internet for health information [51]. A study of transgender and gender diverse people found no interactions between digital health literacy and web-based health-seeking behaviors [29].

#### eHealth Resource Engagement

Ten studies (10/36, 27.8%) examined associations between digital health literacy and eHealth resource engagement with 7 studies reporting positive associations [18,21,31,33,34,36,47]. Higher digital health literacy was associated with signing up for email updates, watching health-related videos, seeking resources from people with similar lived experience, and using health indicator tracking in a general population [47] and among caregivers [34]. Digital health literacy was also associated with willingness to participate in mobile health research interventions, wearing a smart watch or tracking device, and downloading a health app among minority race or ethnicity groups [18,21]. Higher digital health literacy was associated with increased eHealth resource use in older adults [33] and people living with chronic illness [36] and greater use of patient portals in people with chronic disease [47], older adults [33], and transplant recipients [31]. However, no differences were found among varying digital health literacy levels and willingness to participate in research that used web-based forums, support groups, or counseling in an African American sample [18].

#### Access to and Use of Technology

Seven studies (7/36, 19.4%) examined digital health literacy and associations with access to and use of technology with mostly positive associations reported. Access to the internet for personal use was associated with higher digital health literacy among caregivers [35]. Greater internet use was also associated with higher digital health literacy in a low-income population in the United States [52], in Southeast Asians in Canada [21], and in caregivers of children with special needs [34]. Access to and use of digital devices and the internet were associated with higher digital health literacy in an Australian population [16]. Access to any mobile device was also associated with higher eHealth literacy among breast cancer survivors [24]. Furthermore, among older adults, those who owned 2 or more electronic devices had higher digital health literacy than those with 1 or no devices, although no differences in internet use were found across digital health literacy levels [40].

#### Use of a Variety of Health Information Sources

Two studies (2/36, 5.6%) examined digital health literacy and the use of a variety of health information sources. High digital health literacy was associated with the use of more health information sources compared with those with low digital health literacy among older adults [40] and among Black or African Americans [18]. Health information sources included the internet, health books and magazines, TV programs, literature in medical offices, and discussions with health care providers [40]. This study also probed the relationship between digital health literacy and the sources of information used. People with high digital health literacy were more likely to rely on doctors’ knowledge for medical decision making and drew upon more sources of health information than those with low digital health literacy [40]. Similarly, among Black or African Americans, higher digital health literacy was also significantly associated with citing the internet, nurses, books, radio, or news apps as sources of health information [18].

#### Confidence or Comfort With Using Digital Resources

Two studies (2/36, 5.6%) examined confidence in using digital resources and digital health literacy. Older adults with higher digital health literacy were more likely to experience no stress when using a computer than those with lower digital health literacy [40]. By contrast, among population groups digital health literacy was not associated with comfort in using the internet [25].

#### eHealth Satisfaction and Trust

Three studies (3/36, 8.3%) examined digital health literacy and associations with eHealth satisfaction and trust with positive outcomes reported. Higher digital health literacy was associated with greater telemedicine satisfaction among rural and remote communities [22]. Increased digital health literacy was also associated with greater positive perceptions of eHealth in caregivers [34].

In addition, higher digital health literacy was associated with greater perceived trust in eHealth information in a sample of Black or African Americans and Caucasians after controlling for socioeconomic status and social media use [32]. Notably, among individuals with low digital health literacy, older adults had higher perceived trust in Facebook and less trust in support groups than their younger counterparts [32]. Furthermore, in those with low digital health literacy, Black or African Americans were more likely to report greater perceived trust in web-based blogs or diaries and twitter than their younger or Caucasian counterparts; by contrast, for those with high digital health literacy, Black or African Americans were more likely than Caucasians to trust support groups [32].

### Associations Between Digital Health Literacy and Health Outcomes

**Figure 4:**
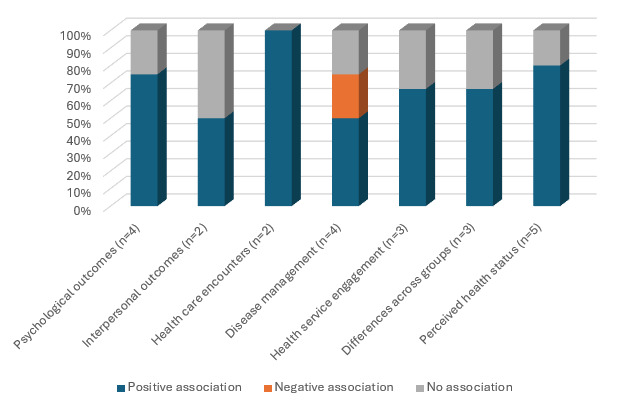
Digital health literacy and behaviors for managing health and chronic disease.

Seventeen studies (17/36, 47.2%) examined associations between digital health literacy and health outcomes. The outcomes are grouped into 3 main categories: psychosocial health outcomes, chronic disease and health management behavioral outcomes, and perceived health status. These are shown in Table 4 and Figure 4 and described in the following sections.

**Table 4:** Digital health literacy and health outcomes.

Category and subcategory of outcomes, and association with digital health literacy				Articles in which the association occurred, n									
				Patient group	General population		Minority group		Older adults		Caregivers		Total
**Psychological**													
	**Psychological**												
		Positive	—^a^		1	—		2		—		3	
		None	—		—	—		—		1			
	**Interpersonal**												
		Positive	—		—	—		—		1		1	
		None	—		—	—		—		1		1	
	**Satisfaction with health care encounters**												
		Positive	—		—	—		2		—		2	
**Behaviors for managing health or chronic disease**													
	**Disease self-efficacy and self-management**												
		Positive	1		—	1		—		1		3	
		None	—		—	1		—		—		1	
	**Health risk behaviors**												
		Positive	—		—	1		—		—		1	
	**Health service engagement**												
		Positive	—		—	1		1		—		2	
		None	1		—	—		—		—		1	
	**Health status across patient groups**												
		Positive	3		—	—		—		—		3	
**Perceived health status**													
	Positive		2		—	1		1		—		4	
	None		—		1	—		—		—		1	

^a^Not applicable.

#### Psychosocial Health Outcomes

Digital health literacy and its relationship with psychosocial health outcomes included diverse subcategories related to psychological outcomes, interpersonal factors, and satisfaction with health care encounters.

##### Psychological Outcomes

Four studies (4/36, 11.1%) examined associations between digital health literacy and psychological outcomes. Higher digital health literacy was significantly associated with greater empowerment through information seeking [49] and less affective distress [44] in older adult populations. Higher digital health literacy was also related to increased information seeking, which was associated in lower cancer fatalism (ie, inevitable death following a cancer diagnosis) in a general population from the United States [48]. By contrast, no associations were found between digital health literacy and worry among caregivers of children with special needs [34].

##### Interpersonal Outcomes

Two studies (2/36, 5.6%) examined relationships between digital health literacy and interpersonal outcomes. One study found that digital health literacy was significantly associated with the size of social networks for seeking information and support for health decision-making in a sample of caregivers of people with prostate cancer [35]. However, digital health literacy was not associated with social functioning, family relationships, or communication skills among caregivers of children with special needs [34].

##### Satisfaction With Health Care Encounters

Two studies (2/36, 5.6%) examined associations between digital health literacy and health care encounters among older adults. Higher digital health literacy was significantly associated with greater satisfaction [41] and less perceived strain in medical encounters [44] in older populations.

#### Behaviors for Managing Health or Chronic Disease

Digital health literacy and individuals’ management of health or chronic disease were related to (1) disease self-efficacy, disease management, and health risk behaviors; (2) health service engagement; and (3) health status across patient groups.

##### Disease Management and Self-Efficacy

Four studies (4/36, 11.1%) examined digital health literacy and disease management and self-efficacy. Three studies examined associations between digital health literacy and individual management of chronic disease and health conditions, with mixed results. In caregivers of men with prostate cancer, higher digital health literacy was associated with greater likelihood of getting a second opinion, awareness of treatment options, and size of social network for information and support in treatment decision-making [23]. In a study of young men who have sex with men, low digital health literacy was associated with decreased likelihood of evaluating personal risk for HIV/STIs, educating others about HIV/STIs, and getting tested for HIV/STI after completing an HIV/STI education intervention [27]. By contrast, in a sample of transgender and gender diverse people [29], no relationship between self-reported digital health literacy and adherence to human papillomavirus vaccination was found.

In 1 study of people living with COPD, those with higher digital health literacy reported greater self-efficacy with managing their chronic disease [39]. In another study that examined associations between digital health literacy and associations with health risk behaviors, higher digital health literacy was associated with greater HIV transmission risk behaviors (eg, unprotected sexual activities or illicit drug use) among women infected with HIV [50].

##### Health Service Engagement

Three studies (3/36, 17.6%) examined associations between digital health literacy and health service engagement with positive findings reported in 2 studies. Higher digital health literacy was associated with greater number of general practitioner visits through increased searches for health information among older adults [49]. Higher digital health literacy levels were also associated with greater likelihood of attending a physical examination by a physician in the prior 12 months in a Black or African Americans sample [32]. By contrast, 1 study found no associations between digital health literacy and health service engagement in a study of transplant recipients. Participants with low digital health literacy were less likely to have talked with a doctor about injury information than those with higher digital health literacy [31].

##### Differences in Digital Health Literacy Across Patient Groups and Health Status

Three studies (3/36, 17.6%) compared digital health literacy across different patient or health status groups and identified varying levels of digital health literacy. Among low-income pregnant women with gestational diabetes or type II diabetes, those with gestational diabetes trended toward higher digital health literacy than those with type II diabetes [28]. In another study, kidney transplant recipients were found to have higher digital health literacy than liver transplant patients [31]. Another paper [46] found higher digital health literacy levels among people with very severe COPD than among those with less severe COPD. In addition, those with lower lung–specific health-related quality of life also had higher digital health literacy levels [46]. These findings were attributed to those with more severe disease accessing the e-resources more frequently to find strategies and information on how to self-manage their disease [46].

#### Perceived Health Status

Five studies (5/36, 13.9%) examined associations between digital health literacy and perceived health status, with studies broadly reporting positive associations. Higher digital health literacy was significantly associated with higher self-reported health status among veterans with spinal cord injury [38] and among African Americans [18]. Digital health literacy was also associated with better self-care, perceived improved quality of life, and increased health status in a community sample aged between 40 and 93 years [41]. Similarly, higher digital health literacy was associated with higher scores on mobility, self-care and usual activities, and lung-specific health-related quality of life in people with COPD; however, digital health literacy was not associated with generic health-related quality of life in this sample [46]. By contrast, no associations were found between digital health literacy and health status in a sample of older US adults [40].

#### Physical or Neurocognitive Health Outcomes

One study each (2/36, 5.6%) explored digital health literacy on physical [38] or neurocognitive health outcomes [45]. Among veterans with spinal cord injury or disorder, no associations were found between digital health literacy and level or duration of injury [38]. In people with HIV, lower neurocognitive function was moderately associated with lower digital health literacy scores [45].

## Discussion

### Principal Findings

This review advances our understanding of consumer digital health literacy through identifying and synthesizing recent literature that explored digital health literacy and its relationship with sociodemographic characteristics and health outcomes. The findings present mixed results regarding the relationship between digital health literacy and sociodemographic characteristics. However, studies broadly suggested that increased digital health literacy was positively associated with improved health outcomes. Based on the findings, we derive implications for practice in closing the digital health divide.

Education was the most common characteristic associated with digital health literacy. People with higher education levels were more likely to have increased literacy skills to better read and interpret web-based health information. Moreover, education has been shown to be a predictor of use of eHealth resources [16]. Education is considered a major determinant of *health literacy*, since educational levels often influence literacy skills, employment status and income, and, as such, enable access to better living circumstances and access to health care [53]. However, some individuals with higher education levels may still have inadequate health literacy [54]. Examining specific skills and knowledge, rather than sociodemographic characteristics alone, may offer a more comprehensive understanding of this relationship in future [53].

Some studies showed that older individuals were more likely to have lower digital health literacy than their younger counterparts, although other studies found no associations. Research has shown that digital health literacy decreases with age, and this is explained by age-related cognitive changes, decreased vision and hearing, reduced motor functioning, and decreased health status [55]. Notably, in our current review, studies with patient populations found no associations between age and digital health literacy levels, and 1 recent study [36] with older people had higher digital health literacy scores than their younger counterparts. Furthermore, in this study [36] the sample was younger and highly educated, and the findings could be related to increased use of digital products. People with chronic health conditions are likely required to engage with digital health resources to manage their health condition, which may account for their higher digital health literacy levels. Thus, while some findings suggest that older people require additional supports to improve digital health literacy capacities, given the varied outcomes, more in-depth analysis of these relationships between digital health literacy and age is recommended for future research including a full systematic review and meta-analysis. Findings showed predominantly no differences in digital health literacy across gender. While several studies reported that women had higher digital health literacy levels than men, the majority found no associations, suggesting that gender is not a likely predictor of digital health literacy. These findings may be attributed to increased use of electronic devices across populations and the closing gap in education levels between men and women [16].

Of the few included studies that examined ethnicity and digital health literacy, findings were also mixed. While some studies reported higher digital health literacy in White populations than in minority groups [25,35], other studies found no differences across ethnic groups [26,38], or conversely that people from minority groups had higher digital health literacy levels [24,32]. Findings from the current review suggest that ethnicity is not a reliable predictor of digital heath literacy; however, further evidence is needed. In addition, studies on digital health literacy among culturally and linguistically diverse communities were limited to a qualitive study, which was excluded from the current review [56]. Quantitative studies in culturally and linguistically diverse populations that met the study criteria were lacking. Thus, further studies are needed to explore these associations and to identify facilitators and barriers to digital health literacy for people in culturally and linguistically diverse communities.

The majority of studies reported positive associations between digital health literacy and health resource navigation, including higher levels of e-information seeking, e-resource engagement, access and use of technology, engagement with health information sources, eHealth satisfaction and trust, and comfort and use of digital resources. The findings suggest a bidirectional relationship between digital health literacy and eHealth resource use: those with greater access to digital devices and greater use of the internet had higher digital health literacy. It is likely that those with more confidence engaging with digital products will be more inclined to seek out health information in digital formats. The findings suggest that ensuring access to technology, as well as fostering skills to engage with eHealth resources is essential to promoting digital health literacy. Furthermore, for people with limited access to the internet or devices, we recommend providing information in nondigital formats.

Our review found that studies examined associations between digital health literacy and health outcomes including psychosocial outcomes, behaviors for managing health or chronic disease, and perceived health status. Studies showed that higher digital health literacy was associated with greater satisfaction with medical encounters, less perceived strain in medical encounters, increased empowerment, greater social networks for health information, and reduced affective distress and cancer fatalism. In these studies, higher digital health literacy enabled individuals to seek and understand information, which enabled them to feel more empowered to manage their health. However, findings were less consistent for associations between digital health literacy and chronic disease and health management outcomes*.*

Studies of chronic disease populations showed that digital health literacy was associated with greater disease knowledge and increased disease management efficacy, consistent with a prior systematic review [57]. However, while some studies suggested that digital health literacy was associated with increased health decision-making behaviors and health service use, other studies found no associations with health promotion behaviors or health service use.

Our findings support conclusions drawn from existing reviews [8], which identified that more studies are needed in the digital health field to examine whether digital advances are facilitating better outcomes for those with greater skills in using e-resources for health purposes. Furthermore, our findings from the review also highlight gaps in recent evidence on the impacts of digital health literacy on prevalent chronic health conditions (eg, cancer, heart disease, diabetes, stroke, and COPD). Given the increased use of telehealth during the COVID-19 pandemic, and importance of chronic disease self-management, research to understand how digital health literacy influences a person’s capacity to engage with digital health resources to manage their health is needed. Furthermore, research on understanding the impacts of digital health literacy on health outcomes in chronic disease populations is recommended. In addition, only 2 included studies examined digital health literacy in caregiver populations. Caregivers, particularly those of adult care recipients, may have unique health information needs given their role in providing support. Given the evidence gap, we recommend further research on caregivers to identify their digital health literacy needs and how these can be addressed across health settings.

### Study Limitations

Across all studies, digital health literacy was assessed using self-reported measures (eg, eHealth Literacy Scale) rather than assessments of specific digital knowledge and skills. Thus, these perceived digital health literacy skills may not translate to everyday digital health behaviors. Studies also varied in their reports of digital health literacy scores (eg, means vs cutoff criteria), thus limiting comparability across studies. Furthermore, included studies captured digital health literacy levels across a range of participant groups, which limit in-depth understanding of digital health literacy within specific populations. Thus, findings should be interpreted with caution for specific groups. Only bivariate associations were reported in the current review. Thus, nuanced understanding of relationships between digital health literacy and health outcomes and sociodemographic characteristics may be excluded. In addition, the aim of the rapid review was to inform the development and implementation of emerging digital health strategies across community settings in real time. In contrast to a full systematic review, the rapid review approach trades some methodological rigor for efficiency in addressing a critical topic and may therefore be vulnerable to bias. While MeSH terms were not used in the database search to limit outputs to articles that focused on measuring digital health literacy, ~1500 publications were still identified. Finally, the findings were limited to studies conducted from English-speaking countries. Thus, articles that capture key findings of the review may have been excluded. Notwithstanding, we note that the current findings highlight substantial gaps in research pertaining to digital health literacy within English-speaking countries and identify areas for future investigation.

### Conclusions

Findings from this review suggest that sociodemographic characteristics may predict digital health literacy levels in some but not all contexts, but evidence suggests that these are not deterministic. Although in its infancy and with limited evidence, studies show some associations between increased digital health literacy and various improved health behaviors and outcomes. Further investigations of digital health literacy on positive chronic disease outcomes are needed, particularly across underrepresented but key populations, including diverse cultural and chronic disease groups. Empowering individuals with the skills to critically access and appraise reliable health information on digital platforms and devices is vital, given emerging evidence that suggests that those with low digital health literacy seek health information from unreliable sources. Identifying cost-effective strategies to rapidly assess and enhance digital health literacy capacities across community settings thus warrants continued further investigation. Our findings also confirm a warning that those with greatest digital skills may obtain greatest benefit from access to digital health resources and vice versa, with the implication that digital divides (gaps between knowledge of digital skills and access to health information) may become entrenched without specific efforts to overcome such divides. Our review contributes to the global digital health movement by identifying areas that require further investigation. It emphasizes the pivotal role of digital health literature in improving health care outcomes and promoting a more inclusive health care system. Digitalization and digital technologies transform and enhance the delivery of health care services; therefore, digital health literature becomes essential to engage health consumers and empower them to actively participate in their own health care and address health inequalities.

## Appendix

Multimedia Appendix 1 Database search strategies.

Multimedia Appendix 2 Characteristics of included studies (n=34).

## Abbreviations

COPD: chronic obstructive pulmonary disease
PRISMA: Preferred Reporting Items for Systematic Reviews and Meta-Analyses

## Data Availability

Data collection forms and extracted data are available upon reasonable request.

## References

[1] Smith B, Magnani JW (2019). New technologies, new disparities: the intersection of electronic health and digital health literacy. Int J Cardiol.

[2] Mackert M, Mabry-Flynn A, Champlin S, Donovan EE, Pounders K (2016). Health literacy and health information technology adoption: the potential for a new digital divide. J Med Internet Res.

[3] Watts G (2020). Covid-19 and the digital divide in the UK. Lancet Digit Health.

[4] Kim J, Jin B, Livingston MA, Henan MD, Hwang J (2022). Fundamentals of digital health literacy: a review of the literature.

[5] Norman CD, Skinner HA (2006). eHealth literacy: essential skills for consumer health in a networked world. J Med Internet Res.

[6] Dong Q, Liu T, Liu R, Yang H, Liu C (2023). Effectiveness of digital health literacy interventions in older adults: single-arm meta-analysis. J Med Internet Res.

[7] van Kessel R, Wong BLH, Clemens T, Brand H (2022). Digital health literacy as a super determinant of health: more than simply the sum of its parts. Internet Interv.

[8] Neter E, Brainin E (2019). Association between health literacy, eHealth literacy, and health outcomes among patients with long-term conditions. Eur Psychol.

[9] Verma R, Saldanha C, Ellis U, Sattar S, Haase KR (2022). eHealth literacy among older adults living with cancer and their caregivers: a scoping review. J Geriatr Oncol.

[10] Stellefson M, Hanik B, Chaney B, Chaney D, Tennant B, Chavarria EA (2011). eHealth literacy among college students: a systematic review with implications for eHealth education. J Med Internet Res.

[11] Chesser A, Burke A, Reyes J, Rohrberg T (2016). Navigating the digital divide: a systematic review of eHealth literacy in underserved populations in the United States. Inform Health Soc Care.

[12] Choukou MA, Sanchez-Ramirez DC, Pol M, Uddin M, Monnin C, Syed-Abdul S (2022). Covid-19 infodemic and digital health literacy in vulnerable populations: a scoping review. Digit Health.

[13] Yang K, Hu Y, Qi H (2022). Digital health literacy: bibliometric analysis. J Med Internet Res.

[14] Khangura S, Konnyu K, Cushman R, Grimshaw J, Moher D (2012). Evidence summaries: the evolution of a rapid review approach. Syst Rev.

[15] Moher D, Liberati A, Tetzlaff J, Altman DG, PRISMA Group (2009). Preferred reporting items for systematic reviews and meta-analyses: the PRISMA statement. Ann Intern Med.

[16] Cheng C, Elsworth G, Osborne RH (2021). Validity evidence based on relations to other variables of the eHealth literacy Questionnaire (eHLQ): bayesian approach to test for known-groups validity. J Med Internet Res.

[17] Hyde LL, Boyes AW, Mackenzie LJ, Leigh L, Oldmeadow C, Riveros C, Sanson-Fisher R (2019). Electronic health literacy among magnetic resonance imaging and computed tomography medical imaging outpatients: cluster analysis. J Med Internet Res.

[18] James DCS, Harville C (2016). eHealth literacy, online help-seeking behavior, and willingness to participate in mHealth chronic disease research among African Americans, Florida, 2014-2015. Prev Chronic Dis.

[19] Richtering SS, Hyun K, Neubeck L, Coorey G, Chalmers J, Usherwood T, Peiris D, Chow CK, Redfern J (2017). eHealth literacy: predictors in a population with moderate-to-high cardiovascular risk. JMIR Hum Factors.

[20] Cherid C, Baghdadli A, Wall M, Mayo NE, Berry G, Harvey EJ, Albers A, Bergeron SG, Morin SN (2020). Current level of technology use, health and eHealth literacy in older Canadians with a recent fracture-a survey in orthopedic clinics. Osteoporos Int.

[21] Makowsky MJ, Davachi S, Jones CA (2022). eHealth literacy in a sample of South Asian adults in Edmonton, Elberta, Canada: subanalysis of a 2014 community-based survey. JMIR Form Res.

[22] Rush KL, Seaton C, Li E, Oelke ND, Pesut B (2021). Rural use of health service and telemedicine during Covid-19: the role of access and eHealth literacy. Health Informatics J.

[23] Singh G, Sawatzky B, Nimmon L, Ben Mortenson W (2021). Perceived eHealth literacy and health literacy among people with spinal cord injury. Arch Phys Med Rehabil.

[24] Moon Z, Zuchowski M, Moss-Morris R, Hunter MS, Norton S, Hughes LD (2022). Disparities in access to mobile devices and e-health literacy among breast cancer survivors. Support Care Cancer.

[25] Austin DW, Bhola P, Tebble C, Shandley K (2018). Preferences for online mental health services among Australian and Indian samples: a cross-cultural comparison. Psychol Stud.

[26] Schrauben SJ, Appel L, Rivera E, Lora CM, Lash JP, Chen J, Hamm LL, Fink JC, Go AS, Townsend RR, Deo R, Dember LM, Feldman HI, Diamantidis CJ, CRIC Study Investigators (2021). Mobile health (mHealth) technology: assessment of availability, acceptability, and use in CKD. Am J Kidney Dis.

[27] Horvath KJ, Bauermeister JA (2017). eHealth literacy and intervention tailoring impacts the acceptability of a HIV/STI testing intervention and sexual decision making among young gay and bisexual men. AIDS Educ Prev.

[28] Steinberg JR, Yeh C, Jackson J, Saber R, Niznik CM, Leziak K, Yee LM (2022). Optimizing engagement in an mHealth intervention for diabetes support during pregnancy: the role of baseline patient health and behavioral characteristics. J Diabetes Sci Technol.

[29] Pho AT, Bakken S, Lunn MR, Lubensky ME, Flentje A, Dastur Z, Obedin-Maliver J (2022). Online health information seeking, health literacy, and human papillomavirus vaccination among transgender and gender-diverse people. J Am Med Inform Assoc.

[30] Norman CD, Skinner HA (2006). eHEALS: the eHealth Literacy Scale. J Med Internet Res.

[31] Maroney K, Curtis LM, Opsasnick L, Smith KD, Eifler MR, Moore A, Wedd J, Wolf MS, Patzer RE (2021). eHealth literacy and web-based patient portal usage among kidney and liver transplant recipients. Clin Transplant.

[32] Paige SR, Krieger JL, Stellefson ML (2017). The influence of eHealth literacy on perceived trust in online health communication channels and sources. J Health Commun.

[33] Price-Haywood EG, Harden-Barrios J, Ulep R, Luo Q (2017). eHealth literacy: patient engagement in identifying strategies to encourage use of patient portals among older adults. Popul Health Manag.

[34] Sarkar M, Sanders LM, Kelleher KJ, Chisolm DJ (2016). Psychosocial health, e-health literacy, and perceptions of e-health as predictors and moderators of e-health use among caregivers of children with special healthcare needs. Telemed J E Health.

[35] Song L, Tatum K, Greene G, Chen RC (2017). eHealth literacy and partner involvement in treatment decision making for men with newly diagnosed localized prostate cancer. Oncol Nurs Forum.

[36] Morton E, Ho K, Barnes SJ, Michalak EE (2021). Digital health literacy in bipolar disorder: international web-based survey. JMIR Ment Health.

[37] Bailey CE, Kohler WJ, Makary C, Davis K, Sweet N, Carr M (2019). eHealth literacy in otolaryngology patients. Ann Otol Rhinol Laryngol.

[38] Hogan TP, Hill JN, Locatelli SM, Weaver FM, Thomas FP, Nazi KM, Goldstein B, Smith BM (2016). Health information seeking and technology use among veterans with spinal cord injuries and disorders. PM R.

[39] Stellefson ML, Shuster JJ, Chaney BH, Paige SR, Alber JM, Chaney JD, Sriram PS (2018). Web-based health information seeking and eHealth literacy among patients living with chronic obstructive pulmonary disease (COPD). Health Commun.

[40] Arcury TA, Sandberg JC, Melius KP, Quandt SA, Leng X, Latulipe C, Miller DP, Smith DA, Bertoni AG (2020). Older adult internet use and eHealth literacy. J Appl Gerontol.

[41] Seçkin G, Hughes S, Yeatts D, Degreve T (2019). Digital pathways to positive health perceptions: does age moderate the relationship between medical satisfaction and positive health perceptions among middle-aged and older internet users?. Innov Aging.

[42] Escoffery C (2018). Gender similarities and differences for e-health behaviors among U.S. adults. Telemed J E Health.

[43] Park H, Cormier E, Gordon G, Baeg JH (2016). Identifying health consumers' eHealth literacy to decrease disparities in accessing eHealth information. Comput Inform Nurs.

[44] Seckin G, Hughes S (2021). Patient-reported outcomes in a nationally representative sample of older internet users: cross-sectional survey. JMIR Aging.

[45] Woods SP, Sullivan KL (2019). Lower neurocognitive functioning disrupts the effective use of internet-based health resources in HIV disease: the mediating effects of general health literacy capacity. AIDS Behav.

[46] Stellefson M, Paige SR, Alber JM, Chaney BH, Chaney D, Apperson A, Mohan A (2019). Association between health literacy, electronic health literacy, disease-specific knowledge, and health-related quality of life among adults with chronic obstructive pulmonary disease: cross-sectional study. J Med Internet Res.

[47] Madrigal L, Escoffery C (2019). Electronic health behaviors among US adults with chronic disease: cross-sectional survey. J Med Internet Res.

[48] Chung JE, Lee CJ (2019). The impact of cancer information online on cancer fatalism: education and eHealth literacy as moderators. Health Educ Res.

[49] Schulz PJ, Fitzpatrick MA, Hess A, Sudbury-Riley L, Hartung U (2017). Effects of eHealth literacy on general practitioner consultations: a mediation analysis. J Med Internet Res.

[50] Blackstock OJ, Cunningham CO, Haughton LJ, Garner RY, Norwood C, Horvath KJ (2016). Higher eHealth literacy is associated with HIV risk behaviors among HIV-infected women who use the internet. J Assoc Nurses AIDS Care.

[51] Aponte J, Nokes KM (2017). Validating an electronic health literacy scale in an older hispanic population. J Clin Nurs.

[52] Li X (2018). Understanding eHealth literacy from a privacy perspective: eHealth literacy and digital privacy skills in American disadvantaged communities. Am Behav Sci.

[53] Stormacq C, Van den Broucke S, Wosinski J (2019). Does health literacy mediate the relationship between socioeconomic status and health disparities? Integrative review. Health Promot Int.

[54] van der Heide I, Wang J, Droomers M, Spreeuwenberg P, Rademakers J, Uiters E (2013). The relationship between health, education, and health literacy: results from the Dutch adult literacy and life skills survey. J Health Commun.

[55] Jung SO, Son YH, Choi E (2022). E-health literacy in older adults: an evolutionary concept analysis. BMC Med Inform Decis Mak.

[56] De Souza RNA, Butt D, Jethani S, Marmo C (2021). Participatory research methods for investigating digital health literacy in culturally and linguistically diverse communities. Transdisciplinary J Cultur Participation.

[57] Xie L, Zhang S, Xin M, Zhu M, Lu W, Mo PKH (2022). Electronic health literacy and health-related outcomes among older adults: a systematic review. Prev Med.

