# Supplementary material for: Digital Health Literacy and Its Association With Sociodemographic Characteristics, Health Resource Use, and Health Outcomes: Rapid Review
Source: Interact J Med Res. 2024 Jul 26;13:e46888. doi: 10.2196/46888 (PMC11316163; doi:10.2196/46888)
Supplement: Multimedia Appendix 1 [file ijmr_v13i1e46888_app1.docx]

**Multimedia Appendix 1**

Search Strategy: PsycInfo

| S8 | S1 OR S2 OR S3 OR S4 OR S5 OR S6 OR S7 | **Search modes** - Boolean/Phrase |
| --- | --- | --- |
| S7 | TI "digital literac*" OR AB "digital literac*" | **Limiters** - Publication Year: 2016-2022; Exclude Dissertations  **Search modes** - Boolean/Phrase |
| S6 | TI "internet health literac*" OR AB "internet health literac*" | **Limiters** - Publication Year: 2016-2022; Exclude Dissertations  **Search modes** - Boolean/Phrase |
| S5 | TI "internet literac*" OR AB "internet literac*" | **Limiters** - Publication Year: 2016-2022; Exclude Dissertations  **Search modes** - Boolean/Phrase |
| S4 | TI "electronic health literac*" OR AB "electronic health literac*" | **Limiters** - Publication Year: 2016-2022; Exclude Dissertations  **Search modes** - Boolean/Phrase |
| S3 | TI "ehealth literac*" OR AB "ehealth literac*" | **Limiters** - Publication Year: 2016-2022; Exclude Dissertations  **Search modes** - Boolean/Phrase |
| S2 | TI "e-health literac*" OR AB "e-health literac*" | **Limiters** - Publication Year: 2016-2022; Exclude Dissertations  **Search modes** - Boolean/Phrase |
| S1 | TI "digital health literac*" OR AB "digital health literac*" | **Limiters** - Publication Year: 2016-2022; Exclude Dissertations  **Search modes** - Boolean/Phrase |

Search Strategy: CINAHL Complete

| S8 | S1 OR S2 OR S3 OR S4 OR S5 OR S6 OR S7 |
| --- | --- |
| S7 | TI "digital literac*" OR AB "digital literac*" |
| S6 | TI "internet health literac*" OR AB "internet health literac*" |
| S5 | TI "internet literac*" OR AB "internet literac*" |
| S4 | TI "electronic health literac*" OR AB "electronic health literac*" |
| S3 | TI "ehealth literac*" OR AB "ehealth literac*" |
| S2 | TI "e-health literac*" OR AB "e-health literac*" |
| S1 | TI "digital health literac*" OR AB "digital health literac*" |

Search Strategy: Medline

|  | Search Terms | Search Options |
| --- | --- | --- |
| S9 | S1 OR S2 OR S3 OR S4 OR S5 OR S6 OR S7 | **Narrow by SubjectGeographic:** - canada  **Narrow by SubjectGeographic:** - australia  **Narrow by SubjectGeographic:** - united states  **Search modes** - Boolean/Phrase |
| S8 | S1 OR S2 OR S3 OR S4 OR S5 OR S6 OR S7 | **Search modes** - Boolean/Phrase |
| S7 | TI "digital literac*" OR AB "digital literac*" | **Limiters** - Date of Publication: 20160101-20221231; Scholarly (Peer Reviewed) Journals  **Search modes** - Boolean/Phrase |
| S6 | TI "internet health literac*" OR AB "internet health literac*" | **Limiters** - Date of Publication: 20160101-20221231; Scholarly (Peer Reviewed) Journals  **Search modes** - Boolean/Phrase |
| S5 | TI "internet literac*" OR AB "internet literac*" | **Limiters** - Date of Publication: 20160101-20221231; Scholarly (Peer Reviewed) Journals  **Search modes** - Boolean/Phrase |
| S4 | TI "electronic health literac*" OR AB "electronic health literac*" | **Limiters** - Date of Publication: 20160101-20221231; Scholarly (Peer Reviewed) Journals  **Search modes** - Boolean/Phrase |
| S3 | TI "ehealth literac*" OR AB "ehealth literac*" | **Limiters** - Date of Publication: 20160101-20221231; Scholarly (Peer Reviewed) Journals  **Search modes** - Boolean/Phrase |
| S2 | TI "e-health literac*" OR AB "e-health literac*" | **Limiters** - Date of Publication: 20160101-20221231; Scholarly (Peer Reviewed) Journals  **Search modes** - Boolean/Phrase |
| S1 | TI "digital health literac*" OR AB "digital health literac*" | **Limiters** - Date of Publication: 20160101-20221231; Scholarly (Peer Reviewed) Journals  **Search modes** - Boolean/Phrase |

Search Strategy: Embase

| #9 | (#1 OR #2 OR #3 OR #4 OR #5 OR #6 OR #7) AND [english]/lim AND [embase]/lim AND [2016-2022]/py |
| --- | --- |
| #8 | #1 OR #2 OR #3 OR #4 OR #5 OR #6 OR #7 |
| #7 | 'digital literac*':ab,ti |
| #6 | 'internet health literac*':ab,ti |
| #5 | 'internet literac*':ab,ti |
| #4 | 'electronic health literac*':ab,ti |
| #3 | 'ehealth literac*':ab,ti |
| #2 | 'e-health literac*':ab,ti |
| #1 | 'digital health literac*':ab,ti |
