# Supplementary material for: Digital Health Literacy and Its Association With Sociodemographic Characteristics, Health Resource Use, and Health Outcomes: Rapid Review
Source: Interact J Med Res. 2024 Jul 26;13:e46888. doi: 10.2196/46888 (PMC11316163; doi:10.2196/46888)
Supplement: Multimedia Appendix 2 [file ijmr_v13i1e46888_app2.docx]

**Multimedia Appendix 2**

|  | Author/s and year | Country the study was conducted | Study design | Participant type | Participant category | Participant characteristics | | | | | | | | Digital health literacy outcomes | |
| --- | --- | --- | --- | --- | --- | --- | --- | --- | --- | --- | --- | --- | --- | --- | --- |
|  |  |  |  |  |  | Sample size | Mean age (SD; years) | Female, n (%) | Ethnicity-White, n (%) | Ethnicity-Black, n (%) | Asian, n (%) | Other, n(%) | Some college, n (%) | Digital health literacy measure | Digital health literacy Mean Score (SD) |
| 1 | Aponte & Nokes (2017) | United States | Cross-sectional survey | Older Hispanic population | Minority group | 100 | 68 (Range 56-85yrs) | 80 (80%) | - | - | - | 100% Hispanic | 25% | eHEALS | Range 3.2 (1.2) – 4.4 (0.7) over 10 ques. |
| 2 | Arcury, Sandberg & Melius et al. (2020) | United States | Cross-sectional survey | Older Adults (55+) | Older adults | 200 | 55-59 (27.5%)  60-64 (31.5%)  65-69 (15%)  70+ (15%) | 116 (58%) | 80 (40%) | 90 | 1 | 2 indig  26 Latino | 70 (35%) | eHEALS | 28.4 (7.1) Range 8-40 |
| 3 | Austin, Bhola, Tebble et a. (2018) | Australia / India | Cross-sectional survey | General population from Australia and India | General population | 487 | 33.55 (SD=12.2 range 18–78 | 333 (68.4%) | 297 Australians (61%) | - | - | 190 Indian (39%) | 72% | eHEALS | (Indian group: M = 25.99 (SD= 5.81); Australian group: M = 29.30 (SD= 5.76) |
| 4 | Bailey, Kohlet & Makary et al. (2019) | United States | Cross-sectional survey | Otolaryngology patients | Patient / chronic disease | 351 | 18-29 y 69 (19.7) 30-49 y 143 (40.7) 50-69 y 107 (30.5) ≥70 y 32 (9.1) | 248 (70.7%) | - | - | - | - | - | eHEALS | 29.76 ± 5.97 |
| 5 | Blackstock, Cunningham & Haughton et al. (2016) | United States | Cross-sectional survey | HIV infected women | Patients/chronic disease | 63 | 49 | 63(100%) | - | 34(56.7%) |  | 23(38.3%) Hispanic  Multiracial 3(5%) | 39 (61.3%) | eHEALS | No HIV risk Beh (n=27) 11 (40.7%) HIV Risk Beh (n=36) 22 (61.1%) |
| 6 | Cheng, Elsworth & Osborne (2021) | Australia | Cross-sectional survey | Patients | General population | 525 | 56.8 (18.6) | 320 (61%) | - | - | - | Australian (not specified) | Cert or Dip 141 (26.9)  Comp Uni or > 175 (33.3) | eHLQ | Range from 2.43 (0.57) - 2.95 (0.68) per 7 questions |
| 7 | Cherid, Baghdadli, Wall et al. (2020) | Canada | Cross-sectional survey | Adults ≥ 50 years with recent fractures | Patient/chronic disease | 401 | 50-64, 164(41) – 131(50)  65-74 117(29)-89(34)  75+ 120(30)-41(16) | 258 (64) – 169 (65) | Not reported | - | - | - | HS Dip/Pro deg 195 (49) 127 49)  Uni Deg 130 (32) 113 (43) | eHEALS | 29, IQR 24–32 |
| 8 | Chung and Lee (2019) | United States | Longitudinal (3 waves) | General population (United States adults) | General population | 578 | 51.4 (16.1) | 50.7% | 434 (75.1%) | 58 (10.0%) | - | Hispanic  48 (8.3%)  Other  26 (4.5%) | Some college 167 (28.9%)  Bach deg 99 (17.1%  Msts deg 48 (8.3%)  Prof or Doct 25 (4.3%) | Cancer fatalism (adopted from Norman & Skinner) | Moderation effect of edu/EHealth literacy on cancer fatalism. |
| 9 | Escoffery (2018) | United States | Cross-sectional survey | United States Adults aged 18-90 years | General population | 400 | 50.7 (17.1) | 200 (49.5%) | 269 (67.3%) | 76 19.0%) | - | Hispanic  76 (19.1%  Other  55 (13.8%) | Some college 136(34.1%)  College 124(31.1%)  Graduate 65(16.3%) | eHEALS | 29.9 (5.9) |
| 10 | Hogan, Hill & Locatelli et al. (2016) | United States | Cross-sectional survey | Veterans with spinal cord injury or disorder (SCI/D | Patients/chronic disease | 290 | 59.6 (12.2) | 8 (2.8%) | 208 (71.7%) | 57 (19.7%) | - | Hispanic /other  25 (8.6%) | Not reported | eHEALS | 27.3 (7.2) |
| 11 | Horvath & Bauermeister (2017) | United States | Cross-sectional survey | Young men (15-24) who have sex with men | Minority group | 127 | 21 (2.2) | - | 70 (55.1) | - | - | - | More than HS | eHEALS | 17.7 (range 6-24) |
| 12 | Hyde, Boyes & Mackenzie et al. (2019) | Australia | Cross-sectional survey | Patients attending for an outpatient MRI or CT scan, 18 yrs or older | Patients / chronic disease | 256 | 53 (15.0%) | 144 (56.3%) | - | - | - | - | More than HS 98 (43.4%) | eHEALS | Awareness 6.9 (2.0); skills 10.9 (2.9); evaluate 10.0 (3.1) |
| 13 | James & Harville (2016) | United States | Cross-sectional survey | African Americanss | General population | 903 | 37.0 (14.7) | 579 (66%) | - | 903 (100%) | - | - | n/r | eHEALS | 3.6 (1.2) to 4.0 (1.1) |
| 14 | Li (2018) | United States | Cross-sectional survey | Census survey recipients in public housing | Minority group | 238 | 47.01 (13.63) | 175 (75.1%) | 54 (24.77%) | 75 (34.4%) | - | 75 (24.4%)  Hispanic  14 (6.42)  Other | Some college 31.76 (74%)  Assoc deg 7.73 (18%)  Bach deg or higher 6.01 (14%) | e-Health literacy scale 2.0 – 9 items | 29.28 (9.83) |
| 15 | Madrigal & Escoffery (2019) | United States | Cross-sectional survey | United States Adults with and without chronic disease (CD) | General population | 401 | 50.7 (17.1) | 200 (49.9) | 268 (66.8) | 77 (19.2) |  | 56 (14.0) Hispanic 78 (19.5) | Some coll 137(34.3%)  College 124(31.0%)  Grad 66(16.5%) | eHEALS | 29.89 (SD 5.95)  30.66 (SD 6.10) with CD  29.43 (SD 5.81) w/o CD |
| 16 | Makowsky, Davachi & Jones (2022) | Canada | Cross-sectional survey | English- or Punjabi-speaking South Asian adults from Edmonton, Alberta | Minority Group | 301 | 39.9 (14.8) | 166 (55.1%) | - | - | Sikh (212 (70.4%); Hindu: 42 (14%_; | Other 47 (15.6%) | <High School 3 (1)  High School 60 (19.9)  ≥College 238 (79.1) | eHEALS | 29.27 (6.84), Range 8-40 |
| 17 | Maroney, Curtis, Opsanick et al. (2020) | United States | Cross-sectional survey | Kidney transplant (KT) and liver transplant (LT) recipients | Patients/chronic disease | 288 | 52.6 (12.91) | 131 (45.49%) | 187 (64.93%) | 87 (30.21%) | - | 14 (4.86%) | College edu or higher 160 (55.56) | eHEALS | 13.7 (SD n/r) Range 8-32 |
| 18 | Moon, Zuchowski, Moss-Morris et al. (2022) | UK | Cross-sectional survey | Breast cancer survivors | Patients / chronic disease | 1860 | 60.5 (SD =11.3, range 28–100) | 1860 (100%) | 1845 (92%) | - | - | 156 (8%) | TBC | eHEALS | 28.8 (7.34) Range 8-40 |
| 19 | Morton, Ho, Barnes et al. (2021) | Canada | Cross-sectional survey | People with bipolar disorder | Patients / chronic disease | 919 | 36.9 (SD 12) | 716 (77.9%) | 560 (61%) | 40 (4.4) | 152 (16.6) | 166 (18.1%) | Post sec 214 (23.3%  UndGrad 324 (35.3%)  PostGrad 173 (18.8%)  Other 31 (3.4%) | eHEALS | 31.7 (SD 6.3) |
| 20 | Paige, Krieger, Stellefson et al. (2017) | United States | Cross-sectional survey | Diverse sociodemographic groups | General population | 811 | 46.3 (17.2) | 589 (72.6) | 402 (49.6) | 162 (40.3) | - | - | Some coll 122(44.2) – 154(55.8)  Bach Deg 71(42) – 98(58)  Mstrs deg 43(39.1) – 67(60.9)  Adv deg 14(27.5 – 37(72.5) | eHEALS | 30.41 (5.19) |
| 21 | Park, Cormier & Gordon et al. (2016) | United States | Cross-sectional survey | Library users | General Population | 108 | 18-29 4  30-39 13  40-49 20  50-59 34  50-59 34  60-69 25  >70 12  More than ½ over 50 | 56 (51.9) | 51.9 (51.9) | 40 (37.0) | - | 12 (11.1%) | Some coll 25(23.1%  AA/AS 10(9.3%)  BA/BS 18(16.7%)  MstrsDeg 18(16.7%)  Doct Deg 13(12.0%) | eHEALS | 29.27 (5.89) |
| 22 | Pho, Bakken & Mitchell et al. (2022) | United States | Cross-sectional survey | Transgender and gender diverse (TGD/Cisgender) | Minority group | 3258 | 31 | - | 2676 | 60 | 75 | 134 Other | Some coll/2yr deg 688 (21.1%)  4yr coll deg 1156(35.5%)  Mst deg > 1233(37.9%) | HINTS  eHEALS | 35.9 (14.2) |
| 23 | Price-Haywood, Harden-Barrios & Ulep et al. (2017) | United States | Cross-sectional survey | Older Adults | Older adults | 247  (137 Users, 110 nonusers) | 63.4 Users  65.2 Nonuser | 159.7 (64%) | 82% users  61% nonusers | 17% users  31% nonusers | - | - | College 60% users  50% nonusers | eHEALS | 32.9 (4.7 Users  24.7 (8.0 nonusers |
| 24 | Richtering et al. 2017) | Australia | Cross-sectional survey | Patients with Cardiovascular risk | Patients / chronic disease | 453 | 67 (8) | 109 (24.1) | 403 (89) | - | - | - | Tech or Voc train 32(21%) - 58(19%)  Undgrad or Postgrad 68(44%) – 174(58.2%) | eHEALS | 27.2 (6.59) |
| 25 | Rush, Seaton, Li et al. (2021) | Canada | Cross-sectional survey | Adults (19 years or older) living in or near a community rural or remote (e.g. population less than 12,000i ) | Minority group | 279 | 49.45 years (SD=14.66) | 197 (70.6%) | 210 (75.3%) | - | 9 (3.2%)  9 (3.2%) | Indig 17 (6.1%)  Other  43 (15.4%) | Trades cert/dip 124 (44.4%)  Uni degree 101 (36.2%)  Missing 1 (0.4%) | eHEALS | Women (M=30.9)  Men (M=28.4). Scores > for those w/use of telemedicine |
| 26 | Sarkar, Sanders & Kelleher et al. (2016) | United States | Cross-sectional survey | Caregivers of Children with Special Healthcare Needs | Caregivers | 313 | 3 groups (30-40; n=140; 41-60; n=154; 61-70;n=19) | 298 (95.21) | 210 (67.09) | 27.8% | &lt; 1% | 103 (32.91) | Some college & above 197 (62.94) | eHEALS | Males 3.63 (0.70)  females 3.51 (0.88) |
| 27 | Schrauben, Appel & Rivera et al. (2019) | United States | Mixed method | Chronic kidney disease | Patients/chronic disease | 932 | 67.9 (9.3) | 41.5% | 37.9% | 51.4% | **-** | Hispanic 7.6%  Other 3.1% | Coll or > 37.8% | eHEALS 8 ques & mHealth/technology surveys 19 ques | eHEALS0.74 (0.63-0.85) MHealth 0.92 (0.88-0.95) |
| 28 | Schulz, Fitzpatrick& Hess et al. (2017) | Canada | Cross-sectional survey | Older Adults from New Zealand, UK and United States | Older adults | 996 | 59.29 (SD 5.43) | (50.1%, 499/996) | (95.6%, 389/407) UK  84.8% (234/276) NZ  79.6% (249/313) United States | - | - | - | The modal educational level was secondary school (41.5%, 413/996); 1.2% (12/996) of the participants had a lower education, whereas 32.3% (322/996) had attended university | eHEALS | Results measured in pathways. Est link b/n eHealth literacy and use of health care services. |
| 29 | Seckin, and Hughes (2021) | United States | Cross-sectional survey | Older Adults ≥ 60 (web-based panel members) | Older adults | 194 | 60 (27%) (194/710) of the total sample (mean 68.7, SD 7.4). 48.8, SD 16.4) Range 18-93yrs | 107 (55.2%) | 160 (80%) | - | - | 20% | 40% (73/194, 37.6%) of the older respondents had a college degree or higher | eHEALS | 2.53 (0.81) range 1-5 |
| 30 | Seckin, Hughes & Yeatts et al. (2019) | United States | Cross-sectional survey | Community sample aged 40–93 | Older adults | 499 | 57.25 (11.26) | 261 (52.3%) | 392 (78.65%) | - | - | - | Bach deg or higher 195 (39.0%) | eHLS | 2.44 (1.04) |
| 31 | Singh, Sawatzky & Nimmon et al.  (2022) | Canada | Cross-sectional survey | Individuals with spinal cord injury (SCI), 19 years + | Patients / chronic disease | 50 | 49.1 (12.7) | 25 (50%) | - | - | - | - | Post Sec 39 (78%) | eHEALS | 31.6 (5.4) |
| 32 | Song, Tatum, Greene et al. (2017) | United States | Cross-sectional survey | Partners of men with newly diagnosed localized prostate cancer | Caregivers | 142 | 61.4 | 142 (100%) | 112 (79%) | **-** | **-** | African Americal or other30(21%) | Some coll or more 97 (68%)  Patients  HS or less 50 (35%)  Some coll or more 92(65%) | eHEALS | 28.5 (Range 8-40) |
| 33 | Steinberg, Yeh, Jackson et al. (2021) | United States | Pilot study | Low-income pregnant individuals with gestational or type 2 diabetes mellitus | General population | 24 | 32.1 +/-5.3 | 100% | 4 (16.7) | 11 (45.8) |  | Latinx 9 (37.5) | College Grad 5 (20.8) | eHEALS, NVS, DES-SF  PAM | 83.75 IQR 76.25-100.00 |
| 34 | Stellefson, Shuster & Chaney et al. (2018) | United States | Cross-sectional survey | Adults with chronic obstructive pulmonary disease | Patients/chronic disease | 176 | 66.19(9.47) | 89 | 169(96%) | 2(1.1%) | 1(0.6%) | 1(0.6%)  Indig  1(1.2%)  Mixed race/other | Some Coll 83(47.2%)  Coll grad 37(21%)  Postgrad deg 29 (16.5%) | eHEALS | 89(50.6%) |
| 35 | Stellefson et al. (2019) | United States | Cross-sectional survey | Adults with chronic obstructive pulmonary disease | Patients/chronic disease | 174 | 66.06 (9.43) | 88 (50.6%) | 168 (96.6) | 3 (1.7) | 1 (0.6) | 2 (1.2%) | 147 (84.5%) | eHEALS | 3.63 (0.71) |
| 36 | Woods & Sullivan (2019) | United States | Cross-sectional survey | Persons living with HIV disease | Patients/chronic disease | 90 | 45.8 (10.1) | 9(10%) | 49(54.4%) | 18(20%) | - | Hispanic  18(20%)  Other  5(5.6%) | 13.7 (2.3) | eHEALS | 6.1 (2.5) |

n/a=Not applicable; n/r=not reported
